# Barriers to vaccine use in small ruminants and poultry in Tanzania

**DOI:** 10.4102/ojvr.v89i1.2007

**Published:** 2022-08-04

**Authors:** Sitira Williams, Isabella Endacott, Abel B. Ekiri, Mirende Kichuki, Mariana Dineva, Erika Galipo, Vadim Alexeenko, Ruth Alafiatayo, Erik Mijten, Gabriel Varga, Alasdair J.C. Cook

**Affiliations:** 1 Department of Veterinary Epidemiology and Public Health, Faculty of Health and Medical Sciences, University of Surrey, Guildford, United Kingdom; 2 College of Veterinary Medicine and Biomedical Sciences, Sokoine University of Agriculture, Morogoro, Tanzania; 3 Zoetis, Zaventem, Belgium

**Keywords:** vaccine, small ruminant, goat, sheep, poultry, challenges, Tanzania, Africa

## Abstract

Vaccination is an important disease prevention and control measure; however, vaccine adoption by livestock farmers in Tanzania is still low. This cross-sectional study examined the challenges to vaccine use faced by livestock owners and animal health professionals (AHPs) in Tanzania. A questionnaire was administered to 216 households that kept small ruminants and poultry and 19 AHPs’ data were collected electronically via the survey platform Qualtrics, and descriptive statistics were performed. Households with poultry reported vaccinating mostly against Newcastle disease (91.7%), fowl pox (48.1%) and Gumboro disease (37.0%), whilst households with small ruminants reported contagious caprine pleuropneumonia (62.2%), sheep and goat pox (17.1%), foot-and-mouth disease (7.3%) and peste des petits ruminants (7.3%). The households’ decision to vaccinate was mostly influenced by knowledge of diseases (82.4%), disease history on the farm (69.4%) and vaccine price (63.4%). Most households (54.6%) experienced challenges when purchasing vaccines, including high vaccine cost (78.0%), long distance from vaccine source (61.0%) and vaccine unavailability (21.2%). The findings suggest that improving the knowledge of livestock owners regarding the priority diseases and the benefits of vaccination, establishing more vaccine suppliers, improving vaccine distribution and access and training AHPs and households on appropriate vaccine storage and handling are necessary to improve vaccine adoption and ensure vaccine quality and effectiveness.

## Introduction

In Tanzania, as in the rest of sub-Saharan Africa, endemic livestock diseases are an obstacle to livestock production because of direct losses in livestock mortality, morbidity and the impact on the livelihoods of livestock keepers. Newcastle disease (ND) in poultry, contagious caprine pleuropneumonia (CCPP) in small ruminants (goats and sheep), contagious bovine pleuropneumonia (CBPP) and East Coast fever (ECF) in cattle are the diseases most often reported by farmers in Tanzania (Covarrubias, Nsiima & Zezza 2012). To counter the impact of such endemic diseases, vaccines are used in livestock to maintain animal health and improve overall production (Roth & Sandbulte 2021). In small ruminants (goats and sheep), there is an economic benefit to controlling and potentially eradicating key endemic diseases, such as peste des petits ruminants virus (PPR), which causes high morbidity and mortality in goats and sheep (Jones et al. 2016). Beyond disease control, vaccination has the potential to increase animal-source food consumption, improve household income and reduce food insecurity (Knueppel et al. 2010).

Although vaccines are known to prevent livestock production losses and reduce the incidence of disease, vaccine adoption in small-holder livestock production in Tanzania is still low. Small-holder livestock producers face several barriers to vaccine use, and there are many interrelated factors that have been suggested to either promote or constrain the use of vaccines, including access to veterinary services, access to distributors and retailers, geographical location (urban vs. rural) (Covarrubias et al. 2012), cost of vaccine and need for refrigeration in the field (Babiuk & Wallace 2018). Another important factor reflected at the household level is gender imbalances (female-headed compared to male-headed households) (Babiuk & Wallace 2018; Covarrubias et al. 2012).

The barriers to vaccine use are likely to differ by livestock species. In small ruminants, CCPP and PPR cause high morbidity and mortality rates and therefore are of high concern to small-ruminant holders (Mbyuzi et al. 2015). However, high concern has not necessarily equated to high vaccine use. For instance, although vaccination is known to be the most effective form of prevention against major livestock diseases, including foot-and-mouth disease (FMD), an endemic disease across many regions in Tanzania, only 5% of livestock households reported vaccinating against FMD (Casey-Bryars et al. 2018). The unavailability of appropriate vaccines, lack of effective policies on vaccine quality, quality control and beliefs on vaccine efficacy have been cited as blocks to vaccine usage (Casey-Bryars et al. 2018; Railey et al. 2018). Although previous literature suggests there are a multitude of factors that may constrain the uptake of vaccines in ruminants, there is still little known about the constraints to vaccine uptake in small ruminants, specifically.

In poultry, ND is amongst the most prevalent diseases, with a mortality rate of up to 90% – 100%, and it is reported to particularly affect rural and remote areas (Hugo et al. 2017). Despite this, vaccination for ND was reported to be low in a previous study that investigated the barriers underpinning the use of the ND vaccine in Tanzania (Campbell et al. 2018). The reported key factors related to ND vaccination in Tanzania have included flock size, knowing someone who vaccinated, use of traditional medicine (Campbell et al. 2018), local support with vaccination, knowledge about ND signs, previous vaccine use and gender (Campbell et al. 2018, 2019). Although previous literature provides useful knowledge about the challenges to ND vaccine use, it is not clear or known if the same challenges apply to other prevalent key poultry diseases that can negatively impact poultry health and production, such as infectious bronchitis, Gumboro disease, Marek’s disease, *Escherichia coli* and salmonellosis.

The role of gender is also important to consider when investigating barriers to vaccine use in livestock. Gender imbalance in vaccine access has been suggested; for example, access to livestock vaccines and animal health information is limited in North-Eastern Uganda (Yusuf 2013). Women are disproportionately affected by challenges in accessing veterinary services, disease information and veterinary pharmaceuticals (Galiè et al. 2017). Regarding livestock ownership, women are more likely to own poultry and small ruminants, whilst cattle ownership is substantially more male-orientated (Njuki & Sanginga 2013). Livestock ownership can provide a sustainable income, which may be particularly important for improving the livelihoods of rural women (Njuki & Sanginga 2013). It is therefore important to consider gender dynamics in livestock production when identifying ways to reduce gender imbalances and to help increase productivity and income generation for female-headed livestock businesses.

The aim of this study was to explore the challenges to the use of livestock vaccines experienced by small-ruminant and poultry farmers, as well as the challenges in providing veterinary services faced by animal health professionals (specifically animal health or livestock extension officers) in Tanzania. The role of gender in small-ruminant and poultry production with respect to vaccine use was also investigated. Exploring the barriers to the use of livestock vaccines in Tanzania will help inform the development of strategies to increase vaccine adoption by small-ruminant and poultry farmers.

## Methods

### Study population and setting

The target study population comprised household heads (referred to as households) of livestock holdings that kept either poultry and/or small ruminants, as well as animal health or livestock extension officers (here after referred to as animal health professionals) in selected regions of Tanzania. For the purposes of this study and in the context of Tanzania, an animal health or livestock extension officer is defined as a person who holds either a diploma or a certificate in an animal health-related field, works closely with the community and reports livestock diseases within their local community areas to the local government.

### Study design

**FIGURE 1:**
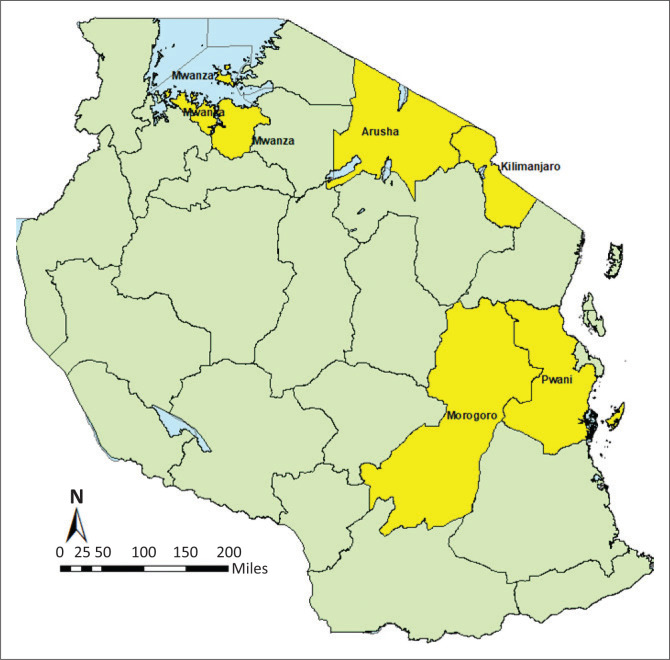
Map of the study regions in Tanzania.

In this cross-sectional study, five study regions across Tanzania were targeted and purposively selected based on the following criteria: (1) areas must have a reasonably large number of small-ruminant and poultry holdings based on the knowledge of key stakeholders and (2) areas must be geographically dispersed sufficiently to capture locational differences regarding the barriers to vaccine use. The following five regions met the above criteria and were thus included in the study: Mwanza (North), Arusha (North-East), Kilimanjaro (North-East), Morogoro (Central) and Pwani and Dar es Salaam (East) (Figure 1). Within each study region, three districts were randomly selected, and within each district, three wards were randomly selected, resulting in a total of nine wards per region and an overall total of 45 wards in the five regions.

Initially, this study was designed to involve focus group interviews targeting a total of 480 study participants, including 450 households (optimally 30 households per district) and 30 animal health professionals selected from the 45 wards. However, because of the coronavirus disease 2019 (COVID-19) international and local travel restrictions and public health guidelines at the time of implementation in June 2020, the study approach was revised to telephone interviews. After revision of the study approach to telephone interviews, the breakdown of the overall total of 480 participants was as follows: (1) one-on-one telephone interviews with households (approximately 450 participants, 10 participants per ward who kept poultry and small ruminants); and (2) one-on-one telephone interviews with animal health professionals (approximately 30 participants from the 45 wards).

To generate contact information for potential participating households, initially, district veterinary officers were contacted and asked to provide contact information for animal health professionals based in each of the selected study wards located in their districts. The animal health professionals were then asked to provide a list of households in their ward, including five households with small ruminants and a minimum of five animals per herd and/or a list of five households with poultry and a minimum of 10 chickens per flock. The determination of the cut-off number of small ruminants or chickens per household was subjective; local animal health professionals were consulted to determine the number of animals kept by a typical household. For the purposes of this study, the households were classified as primarily small-ruminant households or poultry households, based on the number of animals owned; households that owned at least five sheep or goats were classified as small-ruminant households and those that owned at least 10 local chickens or 50 chickens on commercial flock were classified as poultry households.

### Data collection and analysis

Two questionnaires were designed to collect data from household heads of the livestock holdings (Appendix 1 - Material S1) and from animal health professionals (Appendix 2 - Material S2). The questionnaire tools collected data on the following parameters: demographics, livestock holdings, vaccine use, diseases of most concern, prioritised vaccines needed, factors that influence decision to vaccinate, vaccine source, challenges experienced when obtaining or purchasing vaccines and when using vaccines on animals, vaccination campaigns, traditional treatment methods used and the role of gender of small-ruminant and poultry household heads in vaccine use.

The questionnaires were translated to Kiswahili and interviews were conducted in Kiswahili, the most common language in Tanzania, as well as in English. To ensure interpretation was not changed following translation, individuals who were local and fluent in Kiswahili were requested to review the translated questionnaire tools and revisions were made before they were finalised and administered to the study participants. Prior to survey administration, pretesting of the surveys was completed with five households and five animal health professionals, and the questions were revised further for clarity.

A field team of five interviewers was created, which included interpreters knowledgeable in the other local languages. The study team was based and conducted the telephone interviews from a central location in Morogoro. Interview time slots with the participants were scheduled by the field team prior to the interview at a time most convenient for each respondent. Each participant received two reminder calls regarding the date and time of interviews (a week and a day before the interview) with the aims of reducing time-consuming constraints and avoiding delays and rescheduling of the interviews. One-on-one telephone interviews were conducted with the target participants and responses were entered on tablets using an online survey on the Qualtrics platform. Verbal consent was obtained before the start of each interview. Data were collected over a period of 16 days, from 09 November 2020 to 24 November 2020. The data were downloaded from the Qualtrics platform and analysed using descriptive statistics in Microsoft Excel and R (version 1.3.1093).

### Ethical considerations

Ethical review and approval were granted by the Research and Publication Committee of the College of Veterinary Medicine and Biomedical Sciences, Sokoine University of Agriculture (approval date: 14 January 2020).

## Results

There was difficulty in reaching the initially targeted 480 study participants after revision of the study approach to telephone interviews. In the end, a total of 309 households were successfully reached via telephone based on the contact information provided by animal health professionals, and 223 households agreed to participate in the study and were interviewed, providing a response rate of 72.2% (223 out of 309). After excluding seven incomplete interviews, 216 (96.9%) households were included in the analysis. Of the 30 animal health professionals initially contacted, three were not reachable at the time of the study; six of the 27 animal health professionals did not respond to the survey; and of the remaining 21, 19 (90.5%) had complete interviews and were included in the analysis.

### Results from household surveys

#### Respondent characteristics

The largest proportion of the household respondents were from the Pwani and Dar es Salaam region (61 out of 216, 28.2%), followed by Mwanza (48 out of 216, 22.2%), Kilimanjaro (43 out of 216, 19.9%), Morogoro (34 out of 216, 15.7%) and Arusha (30 out of 216, 13.9%). Most of the respondents were men (133 out of 216, 61.6%); the owners of the herd or flock and those in charge of managing the herd or flock were also predominantly men (62.0% and 63.0%, respectively) (Table 1).

**TABLE 1:** Characteristics of the participating household heads (households) (*n* = 216).

Characteristics	Response	Number of respondents	Percent of respondents
Gender (*n* = 216)	Male	133	61.6
	Female	83	38.4
Gender of the owner of the herd or flock (*n* = 216)	Male	134	62.0
	Female	82	38.0
Gender of the individual in charge of managing the herd or flock (*n* = 216)	Male	136	63.0
	Female	79	36.6
	Prefer not to say	1	0.5
Current role, in relation to managing the animals (*n* = 216)	Animal owner	213	98.6
	Manager or supervisor	2	0.9
	Other	1	0.5
Number of people assisting with managing the herd or flock (*n* = 216)	> 2 or less	155	71.8
	> 3–5	54	25.0
	> 5–10	6	2.8
	> Over 10	1	0.5
Number of years worked managing the herd or flock (*n* = 216)	> 1–5	98	45.4
	> 5–10	48	22.2
	> 10–20	49	22.7
	> 20–30	15	6.9
	> 30+	7	3.2
Language used to conduct the survey (*n* = 216)	Kiswahili	145	67.1
	English	41	19.0
	Both English and Kiswahili	25	11.6
	Other (not specified)	5	2.3

Almost all respondents (213 out of 216, 98.6%) were also the actual owners of the livestock holding (Table 1). Nearly half of the respondents had between one and five years of experience in managing their herd or flock (98 out of 216, 45.4%). Most of the telephone interviews with households were conducted in Kiswahili (145 out of 216, 67.1%) (Table 1).

#### Characteristics of the livestock holdings

Poultry households accounted for most respondents (117 out of 216, 54.2%), followed by small-ruminant households (99 out of 216, 45.8%) (Appendix 3 - Table S1). Chickens (174 out of 216, 80.6%), goats (115 out of 216, 53.2%) and cattle (99 out of 216, 45.8%) were the three most common animals kept by the households. Chickens (104 out of 216, 48.1%) were reported to provide the most income for households (Appendix 3 - Table S1). For poultry households (*n* = 117), the most reported average flock size was between 10 and 20 birds (41 out of 117, 35.0%) (Appendix 3 - Table S1). For small-ruminant households (*n* = 99), the most reported average herd size was between 10 and 50 goats or sheep (44 out of 99, 44.4%) (Appendix 3 - Table S1).

#### Vaccine use

**FIGURE 2:**
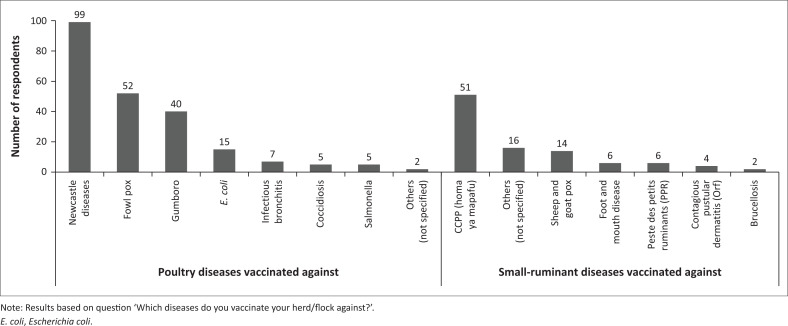
Diseases households vaccinated against in small ruminants and poultry.

Most of the respondents (190 out of 216, 88.0%) had vaccinated their flock or herd in the last 12 months (Appendix 3 - Table S2): 108 poultry households and 82 small-ruminant households. Within those who reported vaccinating, the most common diseases vaccinated against in poultry were ND (99 out of 108, 91.7%), fowl pox (52 out of 108, 48.1%) and Gumboro disease (40 out of 108, 37.0%) (Figure 2), and in small-ruminants they included CCPP (*homa ya mapafu* in Kiswahili) (51 out of 82, 62.2%), sheep and goat pox (14 out of 82, 17.1%), FMD (6 out of 82, 7.3%) and PPR (6 out of 82, 7.3%) (Figure 2; Appendix 3 - Table S2).

#### Diseases of most concern to households and prioritised vaccines needed

**FIGURE 3:**
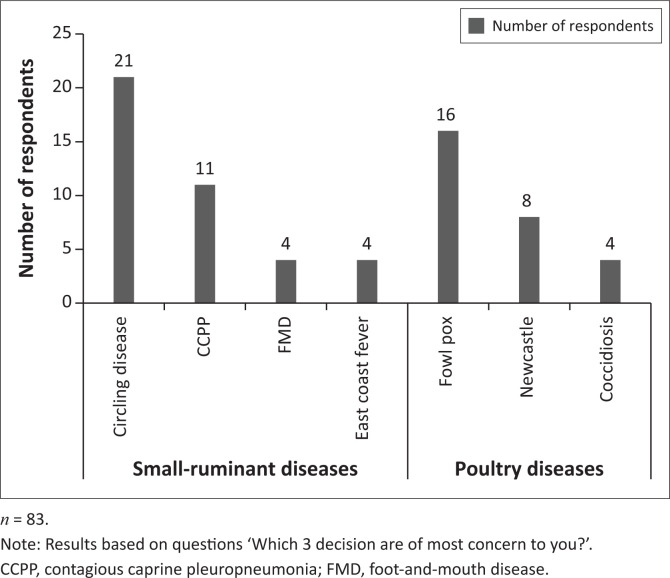
Diseases of most concern to small-ruminant and poultry households.

Over one-third of respondents (83 out of 216, 38.4%) reported that they were concerned about diseases they could not source a vaccine for. The diseases of most concern to small-ruminant households included ‘circling’ disease (actual disease and causative name is not known) (21 out of 40, 52.5%), CCPP (11 out of 40, 27.5%), FMD (4 out of 40, 10.0%) and East Coast fever (4 out of 40, 10.0%) (Figure 3). Poultry households reported fowl pox (16 out of 28, 57.1%), ND (8 out of 28, 28.6%) and coccidiosis (4 out of 28, 14.3%) as the diseases of most concern (Figure 3; Appendix 3 - Table S3).

When households (*n* = 216) were asked about vaccines considered to be of high priority but to which they did not have access, small-ruminant households reported ‘circling’ disease vaccine (17 out of 99, 17.2%) and CCPP vaccine (8 out of 99, 8.1%), whilst poultry households reported fowl pox vaccine (18 out of 117, 15.4%) and ND vaccine (11 out of 117, 9.4%) (Appendix 3 - Table S3).

#### Factors that influence households’ decision to vaccinate

**FIGURE 4:**
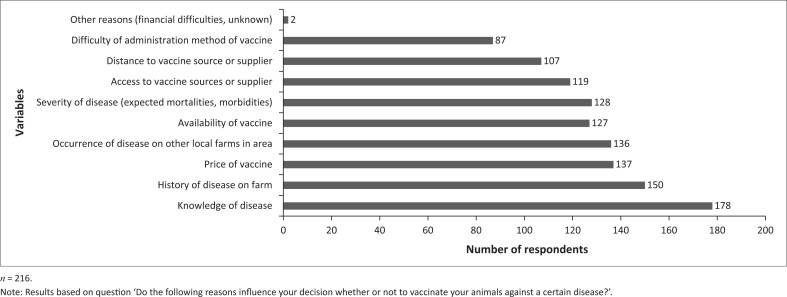
Factors influencing the households’ decision-making to vaccinate their animals.

When respondents were asked about which factors influenced their decision to vaccinate their animals against a certain disease, the most frequently reported factors were the knowledge of diseases (178 out of 216, 82.4%), the history of disease on the farm (150 out of 216, 69.4%) and the price of vaccines (137 out of 216, 63.4%) (Figure 4; Appendix 3 - Table S4). When asked to rank the top two most influential factors in their decision-making, the most frequently reported factors were the price of vaccine (97 out of 216, 44.9%), the knowledge of diseases (54 out of 216, 25.0%) and the distance to vaccine source or supplier (42 out of 216, 19.4%) (Appendix 3 - Table S4). When households were asked about potential consumer-associated drivers and socio-economic factors that would most influence their decision to vaccinate their herd or flock, the three most frequently reported factors were government support with access to expensive vaccines (80 out of 216, 37.0%), information and education regarding administering vaccines safely (58 out of 216, 26.9%) and funding and investment opportunities in agriculture (32 out of 216, 14.8%) (Appendix 3 - Table S4).

#### Vaccine source

Many of the respondents sourced their vaccines from a veterinary or agricultural drug shop (140 out of 216, 64.8%) or directly from a veterinarian or an animal health officer (92 out of 216, 42.6%) (Appendix 3 - Table S2). Most of the respondents travelled between 30 min and 1 h (79 out of 216, 36.6%), followed by under 30 min (71 out of 216, 32.9%) to the source of vaccine purchase (Appendix 3 - Table S2). The animal health officer (115/216, 53.2%) was the most common primary provider of veterinary care and support for the herd or flock, and households predominantly sought advice on vaccine use and vaccination protocols for their herd or flock from a veterinarian or an animal health officer (157 out of 216, 72.7%) (Appendix 3 - Table S2).

#### Challenges experienced by households when obtaining or purchasing vaccines and when using vaccines on animals

Over half of the respondents (118 out of 216, 54.6%) experienced challenges when obtaining or purchasing vaccines for their animals (Appendix 3 - Table S3). Overall, the most reported challenges included the high cost of vaccines (92 out of 118, 78.0%), the long distance from vaccine supplier or source (72 out of 118, 61.0%) and the unavailability of vaccines and/or vaccines not being in stock (25 out of 118, 21.2%) (Appendix 4 - Figure S1; Appendix 3 - Table S3). The most reported challenges when obtaining or purchasing vaccines did not differ by species. Amongst the small-ruminant households (52 out of 99, 52.5%), the three most reported challenges were the high vaccine cost (39 out of 52; 75.0%), the long distance to vaccine supplier or source (28 out of 52; 53.8%) and the vaccines being unavailable or out of stock (13 out of 52, 25.0%). Amongst the poultry households (56.4% (66 out of 117, 56.4%), the three most reported challenges included the high cost of vaccines (53 out of 66; 80.3%), the distance to vaccine supplier or source (44 out of 66; 66.7%) and the unsuitable vaccine package size (14 out of 66; 21.2%).

Almost half of the respondents (100 out of 216, 46.3%) experienced challenges with regard to the use of vaccines on the farm (Appendix 3 - Table S3). Overall, the most reported challenges included problems related to vaccine storage (50 out of 100, 50.0%), ineffectiveness of vaccines against disease (42 out of 100, 42.0%) and vaccine side effects (39 out of 100, 39.0%) (Appendix 4 - Figure S2; Appendix 3 - Table S3). The most reported challenges with regard to the use of vaccines did not differ by species. In small-ruminant households (39 out of 99, 39.4%), the most reported challenges were problems with vaccine storage (15 out of 39, 38.5%), ineffectiveness of vaccine (13 out of 39, 33.3%) and vaccine side effects (13 out of 39, 33.3%). In poultry households (61 out of 117, 52.1%), the three most reported challenges included vaccine storage problems (35 out of 61, 57.4%), the ineffectiveness of vaccines (29 out of 61, 47.5%) and vaccine side effects (26 out of 61, 42.6%).

#### Vaccination campaigns

About one-third of respondents (64 out of 216, 29.6%) reported that their local government had conducted a vaccination campaign in their area in the last 5 years (Appendix 3 - Table S5). Those who reported government vaccination campaigns in their area (*n* = 64) specified that CCPP vaccine (10 out of 64, 15.6%) and rabies vaccine (8 out of 64, 12.5%) were promoted in the vaccination campaigns (Appendix 3 - Table S5). Regarding actual vaccination during the campaign, CCPP (8 out of 64, 12.5%) and anthrax (7 out of 64, 10.9%) were the top diseases vaccinated against in the campaigns (Appendix 3 - Table S5).

#### Traditional treatment methods used by households

To determine what other methods were used by households to treat their herd or flock, the respondents were asked if they had ever used traditional methods to treat animals against diseases or poor health. In total, 44.4% (96 out of 216) of respondents reported that they had used traditional treatment methods for their animals (Appendix 3 - Table S5). Of these, most reported using aloe vera leaves (41 out of 96, 42.7%) or neem leaves (11 out of 96, 11.5%); papaya leaves, moringa and ash were also used by a small proportion of households (Appendix 3 - Table S5).

#### The role of gender of small-ruminant and poultry household heads in vaccine use

To gain further understanding regarding the role of gender in livestock and poultry production across different regions in Tanzania, the respondents were asked whether they considered gender (i.e. being male or female) to be a barrier to vaccine access; only four respondents (4 out of 216, 1.9%), which included two men and two women, agreed with this statement (Appendix 3 - Table S5). When these households were further asked about the ways gender acted as a barrier to vaccine access, the reported reasons included financial opportunities to buy expensive treatment or vaccines (1 out of 4, 25.0%) and that the administration and/or application method of vaccines on their animals was difficult for women (1 out of 4, 25.0%) (Appendix 3 - Table S5).

### Results from survey of animal health professionals

#### Respondent characteristics

The animal health professionals included in this study worked mainly in the Arusha (5 out of 19, 26.3%) and Pwani regions (5 out of 19, 26.3%); the remaining respondents worked in Morogoro (4 out of 19, 21.1%), Mwanza (3 out of 19, 15.8%) and Kilimanjaro (2 out of 19, 10.5%). Animal health professionals were predominantly men (14 out of 19, 73.7%) and the highest level of education attained by most of the respondents was a diploma (13 out of 19, 68.4%) (Appendix 3 - Table S6). Most of the telephone interviews with these respondents were conducted in both English and Kiswahili (7 out of 19, 36.8%) (Appendix 3 - Table S6).

#### Employment characteristics

When the respondents were asked to list the average number of animal health professionals (e.g. animal health officers, veterinarians, para-veterinarians, etc.) who work in each of the ward(s) that they also worked in, the most frequently reported number of animal health professionals per ward was 2 (12 out of 19, 63.2%) (Appendix 3 - Table S6). Most of the respondents reported providing veterinary services to over 20 households on average (12 out of 19, 63.2%) (Appendix 3 - Table S6).

Regarding their current role, most respondents were animal health officers (12 out of 19, 63.2%), and others were livestock extension officers (7 out of 19, 36.8%) (Appendix 3 - Table S6). Most respondents had worked as animal health professionals for 5–15 years (11 out of 19, 57.9%), and the most frequently reported activities they participated in were treating animals (18 out of 19, 94.7%) and advising households on the health and management of their animals (16 out of 19, 84.2%) (Appendix 3 - Table S6). The three most common animal species for which the respondents provided veterinary services (e.g. advice and treatment) in their wards were cattle (18 out of 19, 94.7%), goats (18 out of 19, 94.7%) and sheep (16 out of 19, 84.2%) (Table 2).

**TABLE 2:** Vaccine use, vaccine source and average travel time to vaccine source for households, as reported by animal health professionals (*n* = 19).

Vaccination parameters	Response	Number of respondents	Percent of respondents
Vaccination adoption in poultry flock (*n* = 19)	Yes	17	89.5
	No	2	10.5
Average poultry flock size vaccinated by poultry households (*n* = 17)	Less than 50	5	29.4
	Over 500	5	29.4
	201–500	4	23.5
	51–200	3	17.6
	NA	2	11.8
Vaccination adoption in small-ruminant herds (*n* = 19)	Yes	15	78.9
	No	4	21.1
Average herd size vaccinated by small-ruminant households (*n* = 15)	Over 50	8	53.3
	Less than 5	3	20.0
	21–50	2	13.3
	11–20	1	6.7
	6–10	1	6.7
	NA	4	26.7
Animals receiving veterinary services (e.g. advice, treatment) (*n* = 19)†	Cattle	18	94.7
	Goats	18	94.7
	Sheep	16	84.2
	Chickens	14	73.7
	Pigs	11	57.9
	Rabbits	8	42.1
	Ducks	7	36.8
	Other (not specified)	7	36.8
	Donkeys	1	5.3
Diseases most commonly vaccinated against by poultry households (*n* = 17)†	Newcastle disease	14	82.4
	Fowl pox	13	76.5
	Gumboro disease	8	47.1
	Coccidiosis	4	23.5
	Infectious bronchitis	3	17.6
	*E. coli*	3	17.6
	Salmonella	2	11.8
	Other (not specified)	1	5.9
Diseases most commonly vaccinated against by small-ruminant households (*n* = 15)†	CCPP (*homa ya mapafu*)	13	86.7
	Sheep and goat pox	7	46.7
	Other (not specified)	6	40.0
	Foot and mouth disease	4	26.7
	Brucellosis	1	6.7
	Peste des petits ruminants	1	6.7
Vaccine source for poultry and small-ruminant households (*n* = 19)†	Veterinary or agricultural drug shop	14	73.7
	Directly from a veterinarian, AHO or paravet	11	57.9
	Government provides	7	36.8
	Veterinary pharmaceutical distributor	3	15.8
Average travel time to vaccine source for poultry and small-ruminant households (*n* = 19)	30 min – 1 h	6	31.6
	Over 2 h	6	31.6
	Under 30 min	4	21.1
	1–2 h	2	10.5
	N/A	1	5.3

CCPP, contagious caprine pleuropneumonia; *E. coli, Escherichia coli*; AHO, animal health officer; N/A, Not applicable.

† , Respondents could select all options that apply.

#### Vaccine use

Most animal health professionals reported that poultry households in their ward typically vaccinated their poultry flocks (17 out of 19, 89.5%), and the average poultry flock size vaccinated was mostly less than 50 birds (5 out of 17, 29.4%) or over 500 birds (5 out of 17, 29.4%) (Table 2). Overall, the most common poultry diseases vaccinated against were ND (14 out of 17, 82.4%), fowl pox (13 out of 17, 76.5%) and Gumboro disease (8 out of 17, 47.1%) (Table 2).

Most animal health professionals reported that small-ruminant households in their ward vaccinated their herds (15 out of 19, 78.9%), and the average herd size vaccinated was mostly over 50 animals (8 out of 15, 53.3%) (Table 2). The most common diseases vaccinated against by small-ruminant households were CCPP (*homa ya mapafu*) (13 out of 15, 86.7%), sheep and goat pox (7 out of 15, 46.7%) and FMD (4 out of 15, 26.7%) (Table 2).

#### Diseases of most concern to households and prioritised vaccines needed in ward(s)

About half of animal health professionals (9 out of 19, 47.4%) reported that there were small-ruminant diseases of concern to households and for which they were currently unable to source a vaccine (Appendix 3 - Table S7). The respondents reported that CCPP (5 out of 9, 55.6%), PPR (5 out of 9, 55.6%), FMD (2 out of 9, 22.2%) and circling disease (2 out of 9, 22.2%) were the main small-ruminant diseases households were most concerned about (Appendix 4 - Figure S3; Appendix 3 - Table S7). When animal health professionals (*n* = 9) were asked to report at most three priority vaccines that small-ruminant households in their ward(s) could not access at the time, PPR (4 out of 9, 44.4%) and CCPP (3 out of 9, 33.3%) were most frequently reported (Appendix 4 - Figure S3).

Similarly, about half of animal health professionals (9 out of 19, 47.4%) reported that there were poultry diseases of concern to households and for which they were currently not able to source a vaccine (Appendix 3 - Table S7). Coccidiosis (2 out of 9, 22.2%), Marek’s disease (2 out of 9, 22.2%), typhoid (2 out of 9, 22.2%) and ND (2 out of 9, 22.2%) were reported as the top poultry diseases of most concern to households (Appendix 4 - Figure S4; Appendix 3 - Table S7). Gumboro (2 out of 9, 22.2%), ND (2 out of 9, 22.2%) and typhoid (2 out of 9, 22.2%) were the most reported vaccines when respondents were asked to list the top three priority vaccines that the poultry households could not access at the time (Appendix 4 - Figure S4).

#### Factors reported by animal health professionals that influence households’ decision to vaccinate

Given the experience of the animal health professionals in veterinary diagnostic services, they were asked to give their opinion on the factors that influenced the decisions of poultry and small-ruminant households in their ward(s) to vaccinate their animals against a certain disease. The most frequently reported factors were knowledge of the disease (17 out of 19, 89.5%), history of the disease on farm (16 out of 19, 84.2%) and the occurrence of the disease on other local farms in the area (15 out of 19, 78.9%) (Appendix 3 - Table S8). When asked to select the two main factors, the price of vaccines (8 out of 19, 42.1%), the availability of vaccines (4 out of 19, 21.1%) and the distance to the vaccine source or supplier (4 out of 19, 21.1%) were most frequently reported (Appendix 3 - Table S8). Most respondents stated that animal health officers (7 out of 19, 36.8%) and livestock extension officers (5 out of 19, 26.3%) were most used by poultry or small-ruminant households for veterinary support (Appendix 3 - Table S8).

#### Vaccine source

Most respondents reported that poultry or small-ruminant households sourced vaccines from veterinary or agricultural drug shops (14 out of 19, 73.7%) or directly from a veterinarian or an animal health officer (11 out of 19, 57.9%) (Table 2). The majority of respondents reported that poultry or small-ruminant households in their ward(s) travelled between 30 min and 1 h (6 out of 19, 31.6%), followed by under 2 h (6 out of 19, 31.6%), on average, to the source where they could purchase their vaccines (Table 2).

#### Challenges experienced by households when obtaining or purchasing vaccines or when using vaccines on animals, as reported by animal health professionals

Most animal health professionals (17 out of 19, 89.5%) reported that poultry and small-ruminant households experienced challenges when obtaining or purchasing vaccines for their animals, and the most frequently reported challenges included the distance from vaccine supplier or source (10 out of 17, 58.8%), the high cost of vaccines (8 out of 17, 47.1%) and vaccines not being available or not in stock (7 out of 17, 41.2%) (Appendix 4 - Figure S5; Appendix 3 - Table S9).

Most animal health professionals (13 out of 19, 68.4%) also reported that poultry and small-ruminant households experienced challenges in relation to the use of vaccines on their animals, and the most frequently reported challenges included vaccine side effects (9 out of 13, 69.2%), problems related to vaccine storage (6 out of 13, 46.2%) and the ineffectiveness of vaccine against disease (5 out of 13, 38.5%) (Appendix 4 - Figure S6; Appendix 3 - Table S9).

#### Additional challenges faced by households and animal health professionals

The animal health professionals were asked about other challenges or constraints experienced by poultry or small-ruminant households in relation to the health and production of their animals (i.e. other than vaccine-related challenges). The responses were categorised into themes to allow for meaningful interpretation. The most common responses from the animal health professionals were ‘lack of service providers’ (8 out of 19, 42.1%), ‘lack of education and knowledge’ (5 out of 19, 26.3%) and ‘lack of equipment and facilities’ (5 out of 19, 26.3%) (Appendix 3 - Table S10).

The animal health professionals were also asked about the challenges they experienced in the provision of veterinary services to small-ruminant or poultry households, and the most reported challenges were the lack of tools and equipment for use in clinical work (14 out of 19, 73.7%), insufficient number of animal health professionals in their ward(s) (13 out of 19, 68.4%) and poor road and transport access to households (13 out of 19, 68.4%) (Appendix 3 - Table S10).

#### Vaccination campaigns

Most animal health professionals reported that their local government had conducted a vaccination campaign in their wards in the last five years (11 out of 19, 57.9%) (Appendix 3 - Table S11). When these respondents (*n* = 11) were asked to list the vaccines promoted in the campaigns, the most mentioned vaccines were rabies (5 out of 11, 45.5%), CCPP (4 out of 11, 36.4%), anthrax (2 out of 11, 18.2%) and ND (2 out of 11, 18.2%) (Appendix 4 - Figure S7; Appendix 3 - Table S11). Additionally, based on the responses of the animal health professionals (*n* = 11), the most mentioned diseases vaccinated against in the campaigns were rabies (5 out of 11, 45.5%), CCPP (4 out of 11, 36.4%) and contagious bovine pleuropneumonia (3 out of 11, 27.3%) (Appendix 4 - Figure S7).

#### Traditional treatment methods used on the ward(s)

Most animal health professionals (12 out of 19, 63.2%) reported that poultry or small-ruminant households in their ward(s) used traditional methods to treat against diseases or poor health of their herd or flock; the most frequently reported traditional treatment methods used included aloe vera leaves (used for chickens) (7 out of 12, 58.3%) and neem leaf (6 out of 12, 50.0%) (Appendix 3 - Table S11).

#### The role of gender in vaccine adoption on the ward(s)

When animal health professionals were asked whether they considered gender to be a barrier to vaccine access on their wards, only one respondent (1 out of 19, 5.3%) reported that this was the case, and the reported reason for this was that ‘males tend to leave all farmer managerial activities for the females’ (Appendix 3 - Table S11).

## Discussion

### Vaccine use

Most poultry and small-ruminant households reported to have vaccinated their livestock against at least one disease in the last 12 months, and this information was corroborated by the animal health professionals. Although most respondents reported vaccinating against at least one disease in the last 12 months, the proportion that vaccinated against diseases considered of most concern to small-ruminant and poultry households was extremely low. This finding is significant because vaccination is considered one of the most important preventive measures in disease prevention and control. The results suggest that there is a need for improvement in vaccination adoption through strategies such as increasing awareness of vaccines and related benefits to livestock owners. In addition, it is important to address the reported challenges faced by households related to access and availability of good-quality vaccines, because vaccines considered to be of high priority could not be accessed by households. The involvement of local leaders in efforts to improve vaccine use may also contribute to the success of vaccination drives, as it fosters the feeling of local ownership of the programme and may prevent conflicts with other development activities within the community villages (Msoffe et al. 2010).

### Factors that influence the decision of households to vaccinate their animals

The current study suggests that knowledge is an important component of a strategy aimed at improving vaccine adoption. The factors reported to influence the decision of households to vaccinate animals included knowledge of the disease, history of the disease on farm and vaccine price. Knowledge of the disease and price of vaccine were also ranked amongst the top two most influential factors. In addition, in the current study, about one-third of the respondents reported that their local government had conducted a vaccination campaign in their area within the last five years. This practice may have contributed positively to vaccine adoption in that community, as indicated by the high number of households that reported vaccinating their livestock against at least one disease in the last 12 months. Two previous studies conducted in Tanzania also suggested that knowledge was relevant in vaccination adoption. A previous study reported that knowing someone who vaccinated increased the odds of a household vaccinating, suggesting that provision of relevant knowledge to the community may be a useful strategy to increase vaccine adoption (Campbell et al. 2018). Another study reported that previous vaccine use, gender and support in the village were important factors and recommended that strategies to improve vaccine uptake needed to go beyond simply making vaccines available (Lindahl et al. 2019). Improving awareness of the diseases of concern, the relevant target priority vaccines and the related benefits of vaccinating livestock against disease amongst small-ruminant and poultry farmers may help increase vaccine uptake.

### Challenges related to vaccine access and use experienced by households

High cost of vaccines, the long distance to vaccine suppliers or sources and vaccines not being available and/or not being in stock were the most common challenges faced by households when obtaining or purchasing vaccines. Not surprisingly, the high cost of vaccines and the long distance to vaccine suppliers or sources were also ranked amongst the top factors that influenced the decision of households to vaccinate animals. The reported challenge of the high cost of vaccines highlights the importance of household income as a key driver of vaccine use. Cost of vaccines and distances to vaccination points were also reported as barriers to vaccine uptake in a study in ruminants in neighbouring Uganda and Kenya (Mutua et al. 2019). A previous study in Tanzania that examined poultry farmers’ preferences and ‘willingness to pay’ reported that farmers’ ‘willingness to pay’ was positively influenced by observation of the benefits of vaccination from previous vaccination events (Campbell et al. 2019). Improving awareness of the benefits of vaccinating against disease through vaccination awareness campaigns may help improve a farmer’s ‘willingness to pay’ and consequently vaccine uptake. The challenges of long distances to suppliers or sources combined with the unavailability of vaccines suggest that there are few vaccine sources, and distant geographic location from the vaccine sources might constrain vaccine access and consequently vaccine use. The establishment of more vaccine suppliers, including retailers and distributors which are more geographically dispersed, may help shorten the travelling distances and thus improve vaccine distribution and access, as well as vaccine adoption.

The most common challenges to vaccine use on the farm reported by households and animal health officers were vaccine storage, ineffectiveness of vaccines and vaccine side effects. Although the reasons for the ineffectiveness of vaccines were not investigated in this study, these findings suggest that there is a need to train both animal health officers and households on appropriate vaccine storage and handling to ensure that the quality and the effectiveness are not compromised before administration to the animals. Awareness of the potential vaccine side effects is equally important for minimising misinformation and resentment of vaccine uptake.

The most common animal health and production-related challenges faced by households were the lack of service providers, lack of knowledge and the lack of equipment and facilities, whilst those faced by animal health officers were the lack of tools and equipment to use in clinical work, an insufficient number of animal-health professionals in their ward(s) and poor road and transport access to farmers. These findings highlight potential weaknesses in veterinary care infrastructure and service delivery. Addressing these challenges requires input from both the public and private sectors including the government and animal industry.

The study findings suggest that traditional methods are considered an important option in the treatment of animal diseases. Over two-thirds of household respondents reported using traditional treatment methods for their animals, and of these, most reported using aloe vera leaves or neem leaves. This finding was corroborated by animal health officers, who reported that households used traditional methods to treat their animals. The use of traditional medicine alone and/or in conjunction with Western medicine to manage livestock diseases or conditions is well known. A previous study amongst poultry farmers in Tanzania reported that the use of traditional medicine was associated with a decrease in the likelihood of awareness of ND vaccines and of previous vaccination (Campbell et al. 2018); this suggests that traditional medicine may have a competitive relationship with vaccination. It is important to note that a link (treatment effect) between traditional methods and disease or condition treated was not investigated in this study. Further investigations are required to determine the impact of the use of traditional methods on vaccine uptake, to identify and link the specific traditional method or type of plants and the conditions or diseases targeted and to explore the potential effectiveness of such treatments.

### Role of gender in small-ruminant and poultry production

Findings from the current study indicate that women were less involved in activities and decision-making related to small-ruminant and poultry production. When the few respondents who considered gender a barrier to vaccine access (four households and one animal health professional) were asked about the reasons for this, households reported that the purchase of treatments and vaccines was expensive and the vaccine administration and/or application method was difficult for women. The animal health professionals reported that men tended to leave all farmer managerial activities for the women. These results corroborate the findings of a previous study that also reported a gender disparity in animal health and production roles in Tanzania (Njuki & Sanginga 2013). Gender disparity amongst men and women in livestock production is reported to be highly associated with ownership of land; women in Tanzania have less access and control of land and resources in comparison to their male counterparts (Ndiyo & Urassa 2003; Acosta et al 2022). In addition, women’s involvement in livestock production activities in Tanzania is limited, and women are more likely to be involved in management and administration responsibilities (Campbell et al. 2018). The reported gender imbalance in ownership of livestock can potentially affect food security, as well as women’s access to veterinary services, disease information and veterinary pharmaceuticals (Galiè et al. 2017). Although there are laws already in place for women’s rights to attain land, training and campaigning around equal ownership of land and access to livestock production services and resources are still vital to help women grow, develop and increase their livestock (Idris 2018; Lyimo-Macha & Mdoe 2002).

### Limitations

The change in study approach from focus groups and ethnographic interviews to telephone interviews because of the COVID-19 pandemic disruptions may have impacted the quality of the study findings, as in-depth interviews could not be performed. We could not observe the commonalities and differences between the respondents and explore the responses given, which would have been possible through conducting focus groups. In addition, it was more time-consuming to organise and conduct the interviews; however, given the challenges, this approach was more appropriate for reaching a broader target audience, considering that the respondents were geographically dispersed.

The number of animal health professionals recruited for the study was low; this could be related to the lack of service providers or animal health professionals in the targeted wards, as reported by the animal health professionals who participated in the current study. Because of the small sample size of animal health professionals, the findings from the survey in this study group cannot be generalised to all animal health professionals in Tanzania.

The meaning of the survey questions following translation into Kiswahili may have affected respondents’ interpretations of the questions. For example, the findings might have been affected if questions were misunderstood and answered incorrectly. To minimise this risk and ensure interpretations were not changed, individuals who were local and fluent in Kiswahili were requested to review the translated questionnaire tools, and revisions were made before the tools were finalised and administered to study participants.

Telephone network issues during data collection might have also affected the quality and completeness of the collected data. For instance, some responses may have been rushed, as a handful of farmers were using their own mobile data to participate in the survey and were concerned about the length of time taken to complete the survey. Additionally, some interviews were delayed, paused or rescheduled when network issues were experienced.

## Conclusion

Although most households vaccinated their livestock against at least one disease in the last 12 months, the results suggest that there is a need for improvement in vaccination adoption. As in previous studies, the current study suggests that awareness of the diseases of concern and knowledge of the target priority vaccines and the related benefits of vaccinating livestock against disease should be considered together with other relevant strategies aimed at improving vaccine adoption.

There is a need to address the challenge of the high cost of vaccines. Improving awareness of the benefits of vaccinating through vaccine awareness campaigns may help improve farmer’s ‘willingness to pay’ and consequently vaccine uptake. The challenges of long distance and unavailability of vaccines may be addressed through the establishment of more vaccine suppliers to shorten the travelling distances and improve distribution and access to vaccines. Such efforts will likely require key inputs from the animal industry, particularly the pharmaceutical industry.

Training of both animal health professionals and households on appropriate vaccine storage and handling is needed. This would ensure that the vaccine quality and effectiveness are not compromised before administration to the animals. Improving access and availability of good-quality vaccines and addressing the weaknesses in the veterinary care infrastructure and service delivery are necessary. Efforts to improve vaccine adoption and use will require a multisectoral collaborative approach that involves several key players, including poultry, sheep and goat owners; veterinary professionals; the pharmaceutical industry; and the local and national governments.

Finally, the findings indicated a gender disparity in activities and decision-making for small- ruminant and poultry production; women were less involved and male-headed households predominated. More work is needed to promote and support women to get involved in livestock production as owners or workers, such as through promotion of animal health information and services at the community level.
